# Seeking justice, equity, diversity and inclusion in pediatric nephrology

**DOI:** 10.3389/fped.2022.1084848

**Published:** 2022-12-12

**Authors:** Patricia Seo-Mayer, Isa Ashoor, Nicole Hayde, Marciana Laster, Keia Sanderson, Danielle Soranno, Delbert Wigfall, Denver Brown

**Affiliations:** ^1^ Division of Pediatric Nephrology, Inova Children’s Hospital, University of Virginia School of Medicine-Inova Campus, Fairfax, VA, United States; ^2^ Division of Pediatric Nephrology, Boston Children’s Hospital, Harvard Medical School, Boston, MA, United States; ^3^ Division of Pediatric Nephrology, Children’s Hospital of Montefiore, Albert Einstein College of Medicine, New York, NY, United States; ^4^ Division of Pediatric Nephrology, University of California Los Angeles Mattel Children’s Hospital, Los Angeles, CA, United States; ^5^ Division of Pediatric Nephrology, University of North Carolina, Chapel Hill, NC, United States; ^6^ Division of Pediatric Nephrology, Indiana University, Indianapolis, IN, United States; ^7^ Division of Pediatric Nephrology, Duke University School of Medicine, Durham, NC, United States; ^8^ Division of Pediatric Nephrology, Children’s National Hospital, George Washington School of Medicine, Washington, DC, United States

**Keywords:** racism, equity, social determinants of health (SDOH), pediatric nephrology, workforce

## Abstract

Inequity, racism, and health care disparities negatively impact the well-being of children with kidney disease. This review defines social determinants of health and describes how they impact pediatric nephrology care; outlines the specific impact of systemic biases and racism on chronic kidney disease care and transplant outcomes; characterizes and critiques the diversity of the current pediatric nephrology workforce; and aims to provide strategies to acknowledge and dismantle bias, address barriers to care, improve diversity in recruitment, and strengthen the pediatric nephrology community. By recognizing historical and current realities and limitations, we can move forward with strategies to address racism and bias in our field and clinical practices, thereby cultivating inclusive training and practice environments.

## Introduction

Sociologist Edwin Lindo defines race as “a socio-politically constructed taxonomy…invented on factors such as perceived skin color and culture, not science or biology”. Yet, inaccurate assumptions about the biological significance of race have permeated medical science at all levels—in the classroom, in the laboratory, in the operating suite, and at the bedside. In 2004, as science became capable of articulating new knowledge about human genetic variation, geneticist Francis Collins asserted that “‘race’ and ‘ethnicity’ are poorly defined terms that serve as flawed surrogates for multiple environmental and genetic factors in disease causation, including ancestral geographic origins, socioeconomic status, education and access to health care. Research must move beyond these weak and imperfect proxy relationships to define the more proximate factors that influence health” (1).

Twenty years later, race and racism continue to impact health inequities. In 2020, the deaths of Ahmaud Arbery, Breonna Taylor, George Floyd, and multiple other victims forced broader acknowledgement of the enduring life-and-death reality of racism. The concomitant COVID-19 pandemic cast a brighter light on how social determinants of health (SDoH) influenced exposure to illness, access to care, and mortality rates (2). As a result, there has been increasing commentary and research focused on anti-racism, justice, equity, and inclusion in medicine.

Racism, defined as a system consisting of structures, policies, practices, and norms that assigns value and determines opportunity based on the way people look or the color of their skin, results in conditions that unfairly advantage some and disadvantage others. Racism exists on many levels: *Systemic racism* represents the ongoing racial inequalities that were set in motion centuries ago and continue to be maintained by our current society. This form of racism infiltrates the social determinants of health leading to inequality in financial opportunity, housing, and ultimately health (3). *Institutional racism* refers to the discriminatory policies and practices within organizations and institutions. An example of this in healthcare is the strategic distribution of high-quality medical institutions away from areas of lower income. *Individual racism* and bias impact direct patient assessment, treatment planning, and adherence (4).

Pediatric nephrologists have a unique role in acknowledging and diffusing the impacts of racism. Children are vulnerable members of society in whom adverse effects of racism and bias can potentially be avoided. Disparities in kidney care, which involves complex and expensive treatment modalities such as dialysis and transplantation, are amplified by racism and social drivers of health. As Mohottige and colleagues assert, “an understanding of race and racism is integral to kidney care” (5). Pediatric nephrologists bear witness to how systemic, institutional, and individual racism impact social determinants of health, including housing, neighborhood, education and literacy opportunities, employment, and access to care. Racism and bias also impact biochemical and hormonal pathways that affect health outcomes. Bignall and Crews recently called the kidney community to arms: “We must now use our collective scholarship and expertise to put kidney health equity into action” (6).

To be fully equipped to take on this effort, it is imperative that we educate ourselves. In 2022 at their annual meeting, the American Society of Pediatric Nephrology (ASPN) convened a series of lectures designed to educate the pediatric nephrology community on the most recent and impactful data describing barriers to equitable care, the impact of SDoH and racism on pediatric CKD and transplant outcomes, and the state of the current pediatric nephrology workforce through the lens of diversity, equity and inclusion. A roadmap of curricular resources and next directions was also presented. This review summarizes salient points communicated during those lectures.

## Social determinants of health: Real barriers to receiving equitable care

*“When it comes to health, your zip code is more important than your genetic code.”—Dr. Garth Graham (* *7* *).*

In 400 BC, Hippocrates observed that poor environmental settings are bad for health (8), a truth that remains today. SDoH are the conditions in which people are born, grow, live, work, and age that affect health and contribute to health inequities. The United States Department of Health and Human Services categorizes SDoH into 5 key domains: education, social and community context, economic stability, neighborhood/built environment, and health/healthcare (9). These factors are shaped by the distribution of money, power, and resources at global, national, and local levels (10).

The World Health Organization (WHO) convened a Commission on SDoH in 2008 and called for a three-tiered approach: (1) Improve daily conditions, (2) Tackle the inequitable distribution of power, money and resources, and (3) Measure and understand the problem and assess the impact of action. The American Academy of Pediatrics (AAP) issued a policy statement in 2016 entitled “Poverty and child health in the US,” which urged pediatricians to routinely screen for SDoH. In 2019, an AAP survey found that 62% of pediatricians felt that SDoH screening was important, however, only 39% felt it was feasible, and just 20% felt prepared to address patient needs (11). Data from the 2019 National Survey of Healthcare Organizations and System (NSHOS) determined that 24% of hospitals and 15.6% of practices screened for all 5 SDoH (12). While multiple validated tools to screen for SDoH exist, as described by Sokol et al., their use can be limited by language availability and appropriateness for low-literacy populations.

In 2019, Crews and Novick characterized chronic kidney disease (CKD) hotspots: “countries, regions, communities or ethnicities with higher-than-average incidence of CKD when compared with the worldwide, country or regional rates” (13). They and others have proposed potential interventions and supported the need for a conceptual framework emphasizing the importance of socioeconomics as a mediator of key CKD prevention and treatment pathways (14). Studies have identified multiple factors impacting risk of CKD, such as preterm birth, obesity, diabetes, hypertension and endothelial dysfunction, chronic inflammation, neurohormonal activation and oxidative stress, conditions largely rooted in socioeconomic deprivation and its outcroppings or extensions. These include—but are not limited to—discrimination and segregation, substandard living conditions, limited quality health care to the uninsured or underinsured, limited health literacy, poor educational systems and chronic stress. Altogether, this results in measurable and quantifiable pathologic factors that contribute to, and promote the development of CKD, progression to end-stage kidney disease (ESKD), and an increased risk of premature mortality. We will focus on several specific SDoH, namely food insecurity, income, and education, and their impacts on child kidney health.

## Food quality and food insecurity

The role that diet and access to food plays in the causation and prevention of obesity and heart disease has been studied for decades, and food deserts are often seen in lower income areas and where greater numbers of people of color live. Morland et al. examined populations in four states (Mississippi, North Carolina, Maryland, and Minnesota), and discovered an association between the physical availability of food stores and food service businesses and people's adherence to health authorities’ recommendations for a healthy diet (15). Authors observed that 8 times as many Black Americans lived in the lowest-wealth neighborhoods compared to the highest-wealth areas. Furthermore, there were over 3 times as many supermarkets in the wealthier neighborhoods compared to the lowest-wealth areas. Poor neighborhoods had 3 times more places to consume alcoholic beverages (prevalence ratio [PR] = 0.3, 95% confidence interval [CI] = 0.1–0.6).

Food insecurity, defined by the United States Department of Agriculture (USDA) as lack of consistent access to a sufficient quantity of affordable and nutritious food, is linked to increased health care use and expenditure, even when accounting for other socioeconomic factors. Berkowitz et al. determined that food insecurity was associated with higher rates of emergency department visits, hospitalizations, and duration of days hospitalized (16). Living in a food desert impacts one's cardiovascular risk factors, rates of obesity, and rates of developing hypertension and chronic kidney disease. Food insecurity disproportionately affects pediatric nephrology patients: Starr et al. screened a pediatric ESKD dialysis population and determined that 64% had food insecurity. The investigators utilized a validated food insecurity screening tool that asked:

• *“Within the past 12 months [we] worried whether [our] food would run out before [we] got money to buy more”*
• *“Within the past 12 months the food [we] bought just didn't last and [we] didn't have money to get more.”*

In this group, there was an association between food insecurity and number of emergency department visits, unplanned admissions, and a statistically significant link between positive screening for food insecurity and number of infections (17). In response to this data, an in-unit food pantry was established. Children's hospital systems are now developing pathways to screen patients and connect them directly to community organizations in real time, in the context of clinical visits (18).

Income and education. A pediatric subspecialty study in British Columbia determined that in children with CKD, lower level of family income (<$45,000/year) was associated with more rapid decline in glomerular filtration rate (GFR). Moreover, lower caregiver education was associated with poorer health outcomes (19). Montini et al. described that in a Nicaraguan population, children with CKD and very low parental educational status/inability to read were at the highest risk for death, with a Hazard Ratio of 2.73 (20).

Intensive early education of children poses the potential to improve health and social outcomes. The Carolina Abecedarian Project (ABC) was a randomized controlled trial of early education which enrolled infants from 1972 to 1977 at the Frank Porter Graham Child Development Institute in Chapel Hill, North Carolina (21). One-hundred-eleven infants were randomized to receive an intensive early education program or nutritional supplements and parental counseling alone; participants have been followed to the present day. Treated children demonstrated improved cognition in both short and long-term follow-up (22). Compared to controls, members of the ABC treatment group were more likely to graduate from college, 6 times less likely to utilize public assistance, less likely to experience depressive symptoms, had lower average systolic blood pressure (126 vs. 143 mmHg in controls), and were less likely to develop risk factors for cardiovascular and metabolic disease (23).

In summary, SDoHs play a profound role in health outcomes. They intersect with racism and discrimination, and such inequities confer significantly increased morbidity and mortality particularly in Black and Hispanic children. As such, pediatricians and subspecialists should advocate for regular SDoH screening and for creating systems to connect patients to resources, especially when needs could close existing gaps in health inequities. Health care systems, and local and national governments can address SDoH for children by advocating for and supporting greater distribution of funding and access to resources for all children, especially in communities that have historically experienced systematic denial of capital. Studying CKD “hotspots” more closely may prove an additional strategy to implement interventions in the kidney patient population (24).

## The impact of systemic bias and racism on CKD and transplant outcomes

In addition to providing a direct barrier to equitable health services, racism also exerts a biological impact on the sufferer, further contributing to poor health outcomes. The biological effects of racism and discrimination include increased allostatic load which represents the cumulative burden of chronic stress. This manifests as increased sympathetic nervous system activity, altered gene expression and alterations in the metabolism of hormones like cortisol and insulin. This combination contributes to the genesis of disparate disease outcomes. For kidney disease, these mechanisms are directly associated with factors which worsen kidney disease progression including hyperfiltration, inflammation, and renin angiotensin aldosterone system activation (25).

CKD and ESKD disproportionately affect patients of color, with a four-fold increased incidence in Black patients and two-fold increase in Hispanic individuals (26). Compared to White patients, minoritized patients have reduced access to preventative care, delayed referral to nephrology care, poorer hypertension control, and poorer access to diagnostic testing. Once they reach the nephrologist, patients of color have lower rates of high-quality dialysis service, home dialysis, transplant activation, living kidney donation, and transplantation itself (25, 26).

In pediatrics, racial bias has been linked to numerous disparate outcomes in care, even before infants are born, and these impacts can be observed in the pediatric nephrology clinic. Disparities in gestational age and birth weight are of interest to the nephrologist due to the link between nephron endowment and later CKD and hypertension (HTN) risk (27). Burris et al. reported that the risk of preterm birth—a known risk factor for development of CKD and HTN in children—is significantly higher in non-Hispanic Black women as compared to White women, with a relative risk of 52% (28). The increased risk of preterm birth was seen across all levels of maternal education. In fact, the relative risk of preterm birth in the Black college graduate mother was 2.2-fold higher than in White college graduates, indicating disparity in preterm birth was *accentuated* with higher maternal education.

Orchard et al. underscored the connection between health outcomes and the experience of racism in their 2017 study examining prejudice and the association with preterm birth (28). Using results from Implicit Association Tests (29), and questions to determine explicit prejudice, the authors defined counties as more or less prejudiced than the mean. They identified a baseline difference in preterm birth frequency between Black and White births across all levels of prejudice, both implicit and explicit. Additionally, as the level of prejudice increased, the frequency of preterm birth amongst Black women increased, further widening the racial gap in preterm birth risk (30).

A 2011 study by Gutierrez et al. illustrated the impact of SDoH on CKD-relevant outcomes. Fourteen thousand adult participants enrolled in the National Health and Nutrition Examination Survey (NHANES) were evaluated, and in both the unadjusted and fully adjusted models, worsening poverty was associated with increased odds of hyperphosphatemia. The most severely impoverished patients had a 2.2-fold higher odds of hyperphosphatemia as compared to the highest income group. This association was observed despite lower reported phosphate intake within the most impoverished group. This discrepancy may reflect hidden phosphorus sources such as the consumption of phosphorus-rich additives frequently found in highly processed foods (31).

Recent literature details the impact of systemic bias and racism on pediatric nephrology care and outcomes. The Chronic Kidney Disease in Children (CKiD) study demonstrated racial differences in CKD progression. In 110 Black or African-American and 493 non-African American children with non-glomerular CKD, Ng et al. reported a higher rate of decline in GFR per year with children identified as Black or African-American, with a decline of 6.2% per year decline as compared to a decline of 4.3% per year in non-African-American children (32). Of note, controlling for socioeconomic status removed the significance of these differences suggesting differences in socioeconomic status may mediate these differences. Consistent with GFR decline, the median time to kidney replacement therapy in Black children was 3.2 years earlier than non-Black children. Upon adjustment for socioeconomic status, the median time to kidney replacement therapy was still 1.6 years faster for Black children.

Another area of interest is in understanding the role of genetic markers, such as Apolipoprotein L1 (APOL1), on mediating disparities in CKD progression. Parsa et al. recognized that patients of black ancestry were at increased risk for ESKD compared to White patients, and examined the effects of gene variants encoding APOL1 on progression to ESKD (33). Patients from two large cohorts, the African American Study of Kidney Disease and Hypertension (AASK) study, and the Chronic Renal Insufficiency Cohort (CRIC) study, were stratified by number of copies of high-risk APOL1 variants. Black patients enrolled in AASK with 2 copies of high-risk APOL1 variants progressed to ESKD faster than Black patients with one copy, and in the CRIC study, Black patients with 2 high-risk variants had more rapid decline in in GFR compared to White patients (*p* < 0.001). Furthermore, in multivariate analysis, Black patients *without* the high risk APOL1 genotype also had greater risk of GFR decline as compared to White patients. This latter finding suggests that despite promising targets like APOL1 impacting genetic predisposition to ESKD progression, this is only part of the story and does not fully account for observed disparities.

Unfortunately, once patients progress to end stage kidney disease and require dialysis, racial-ethnic differences in survival are further highlighted. Laster et al. analyzed approximately 2,600 children from a large dialysis organization and found a 64% higher risk of mortality in Black children as compared to White children (34). There was no difference in survival between Hispanic and White children although the trend was toward better survival in Hispanic children. This finding is consistent with studies in the adult population and remains unexplained. In the same study, researchers also observed that Black children had 39% lower likelihood of transplantation and Hispanic children had 12% lower likelihood of transplantation.

Pre-emptive transplantation requires early recognition of kidney disease progression and rapid access to pre-ESKD nephrology care. There are known racial-ethnic differences in the receipt of pre-emptive transplant. Patzer et al. collected 9 years of data on living donor transplant in children between 2000 and 2009 (35) and determined that living donor preemptive transplant rates were significantly lower in minority patients as compared to White children. More specifically, pre-emptive transplant rates were 66% lower in Black patients and 52% lower in Hispanic patients.

Not only are minority children less likely to receive a pre-emptive transplant, they are also less likely to receive a living donor transplant in general. Amaral et al. studied 19,722 incident ESKD pediatric patients who received living donor transplants and demonstrated that the overall number of living donor transplants decreased between 2005 and 2015 with rates varying according to racial group. Black children were 62% less likely to receive a living donor kidney transplant (LDKT), Hispanic children were 46% less likely, and children of Asian background were 63% less likely than White children (36). There were disparities in donor-recipient concordance as well; while 95% of non-Hispanic White children were likely to receive a kidney from a non-Hispanic White donor, only 56% of Asian recipients had Asian donors. Socioeconomic factors that allow individuals to step forward as kidney donors are a major contributor (37). Literature also supports that deceased donor transplant rates are lower among Black children compared to Whites (38).

Even after receiving a transplant, survival in Black patients is lower when compared to White patients. Becerra et al. provided a comprehensive view of survival and transplant outcomes by analyzing data from approximately 28,000 participants included in the United States Renal Data System (USRDS) wherein kidney replacement therapy was initiated prior to 18 years of age. Thirty years after the onset of kidney replacement therapy (dialysis or transplant), 39% of Black patients survived as compared to 57% of White patients, indicating a 45% higher risk of mortality for Black patients, even after adjusting for clinical factors, income, and insurance status (38). Black children also had lower incidence of transplant, lower number of transplants, and less time spent with a functioning transplant. If transplant-related factors were equalized, authors calculated that the disparity in mortality would be reduced by 35% through the equalization of transplant access and outcomes. This study posited that while there remains much to be learned about race-based survival differences, equity in transplantation access may play a pivotal role in observed disparities in post-transplant patient survival.

An additional factor crucial to this discussion is that the effects of racism may directly impair patients’ abilities to process and implement medical recommendations. A 2021 study on executive functioning in a multiethnic cohort of 319 college students evaluated the association between recent experiences of discrimination and the components of executive function including working memory, cognitive flexibility and inhibitory control (39). Having an experience of racial discrimination was significantly associated with lower cognitive flexibility, or the ability to switch perspectives and the way one thinks about problems. Recent racial discrimination was also associated with decreased working memory, or the ability to maintain and manipulate information. CKD in children is known to negatively impact intelligence quotient (IQ), academic achievement, attention regulation, and executive functioning. Thus, the presence of CKD compounded by experienced racism and discrimination exerts significant adverse effects on neurocognitive function (40). Ultimately, under-represented and marginalized patients are performing a perpetual juggling act. While physicians attempt complex conversations about medications, immunosuppression, dialysis and transplant, our patients are battling pressing and often competing factors such as access to food and stable housing. The impact of racism on cognitive functioning, in concert with the known effects of CKD on neurocognitive functioning, creates substantial barriers to quality care.

## Diversity in our own backyard: The pediatric nephrology workforce

One potentially modifiable factor protecting minoritized patient populations is physician-patient racial concordance. Physician-patient racial concordance improves patient satisfaction, patient-provider communication and medication adherence (41). In a study of 1.8 million hospital births between 1992 and 2015, Greenwood et al. determined that newborn-physician racial concordance is associated with significant improvement in Black infant mortality. Simply put, “Black physicians systemically outperform their colleagues when caring for Black newborns” (41).

Unfortunately, certain racial and ethnic populations remain under-represented in medicine (URiM). In 2003, the AAMC defined URiM as “those racial and ethnic populations that are underrepresented in the medical profession relative to their numbers in the general population” with the objective to evolve with the changing demographics of society and the medical profession, and focus on regional and local perspectives (42). URiM populations at the current time include African Americans and/or Black, Hispanic/Latino, Native American (Indigenous peoples, Alaska Native, and Native Hawaiian), Pacific Islander, and Mainland Puerto Rican (43); some populations include Filipino, Hmong, and Vietnamese, as well as two or more races, when one or more of the previous listed are represented.

The most recent AMA Physician Masterfile Survey indicated that of 936,254 physicians surveyed in 2019, 63.7% of physicians were male and 36.3% were female (Table 1). The data lacked a “nonbinary” distinction which may further impact the data accuracy, and included a footnote in the report that the survey excluded “2,726 active physicians whose sex is unknown.” Nephrology (no noted distinction between pediatric and adult) was comprised of 71.1% males, and 28.9% females (48). Pediatrics was predominantly female (64.3% female). While no specific data about pediatric nephrology was provided, other pediatric subspecialties show unique gender predilections, with pediatric cardiology being 62.9% male, and peds oncology which was 55.1% female (42).

**Table 1:** Gender and race demographics relevant to the pediatric nephrology workforce.

Group	Source, year published	Gender			Race					
		Male	Female	Non-binary or other categories	White	Black/African-American	Hispanic/Latino	Asian	Multiple or other	Unknown/not reported
US medical school graduates	AAMC (42), 2019	52.1%	47.9%	NR	54.6%	6.2% −44.7% African-Am −16% African −8% Afro-Caribbean −31% “combination of mult subgroups”	5.3%	21.6% −14 different ethnicities reported	8%	
Attending physicians	AAMC, AMA Masterfile (44), 2019	64.1%	35.8%	0.2% “Unknown”	56.2%	5%	5.8%	17.1%		13.7%
Pediatric residents	Montez et al. Pediatrics (45), 2021	29%	71%	NR		5.6%	9.7%		2%	10%
Pediatricians	AAMC, AMA Masterfile (44), 2019	36.2%	63.8%	NR	54.7%	6.2%	7.2%	13.8%	0.9% Multiracial 0.6% Other 0.3% AI or AN 0.1% NH or PI	16.2%
Pediatric nephrologists	Primack et al. Am J Kid Dis (46), 2015	51%	49%	NR	70%	4%	NR	21%	5%	
Pediatric nephrologists (ASPN members)	ASPN annual report (47), not published	29%	58.3%	12.5% Preferred not to answer or left blank	49.4%	4%	2.9%	16.1%	2.4% not specified 17.1% specified as write-ins	21.7%
US population	US Census Bureau, 2016	49.5%	50.5%	NR	51%	15%	24.9%	5.2%		
Projected US population in 2060	US Census Bureau, 2016				36.4%	16%	31.9%	18%		

AAMC, Association of American Medical Colleges; AMA, American Medical Association; ASPN, American Society of Pediatric Nephrology; NR, not reported; AI, American Indian; AN, American Native; NH, Native Hawaiian; PI, Pacific Islander.

The pipeline of US Medical School graduates is similarly disparate in representing the general population. Workforce data from 2018 indicates that only 11.5% of US Medical School graduates are Black/Hispanic (which by definition would encompass URiM) and that Asians represent 21%, though *via* 14 distinct ethnic groups which are not sub-analyzed (42). The percentage of pediatric residents who would be considered URiM is higher (16.5%) than that of the general pool of US medical student graduates, but this figure has not changed substantially since 2007 when it was 16% (45). Similarly, the number of URiM pediatric subspecialty fellows has stagnated at 14% for the past 12 years.

Looking specifically at URiM representation in pediatric nephrology, the sole comprehensive pediatric nephrologist workforce survey in the literature, commissioned by the AAP in 2013 and published by Primack et al. in 2015, characterized the pediatric nephrology workforce *via* email survey to any candidates listed by the American Board of Pediatrics as “board eligible” or “board certified” in pediatric nephrology, members of the ASPN, and members of the AAP Section on Nephrology at the time of the survey. 504 of 766 eligible physicians responded (response rate: 65.8%). Trainees were not included. Although 51% of pediatric nephrologists identified as male, 64% of recent fellowship graduates (<15 years from training) were women (46). Of respondents, 70% were White, 21% identified as Asian, 4% were Black and 5% were “multiple” or “other”. Updated demographic data on the pediatric nephrology workforce was presented in the 2021–2022 ASPN Annual Report (47) and highlights that the number of URiM members is still low, and that the multi-ethnic nature of nephrologists is difficult to capture. Notably, the AAP and ASPN are commissioning an updated workforce survey in 2023, with a focus on gathering more in-depth demographic data on diversity and representation. Regardless, the pipeline of URiM students and residents imparts limitations, indicating that mentoring and recruitment of URiM learners must start sooner (49).

In addition to these data on race in the physician workforce, there are also interesting socioeconomic correlations worth noting as they may contribute to the lack of racial and ethnic diversity in medicine and, subsequently, the pediatric nephrology labor force. One such trend is that the majority (>51%) of 1st year medical students were from the top quintile of US household income, defined as >$225,251, reflecting the top 5% earners (44). Data such as this is likely a reflection of multigenerational inequities, systemic structures of wealth known to dissimilarly impact racial and ethnic groups, and other factors that influence a student's, particularly one from an underrepresented background, access to a pre-medical career path.

## Training the next generation: How do we open the pipeline and diversify our field?

*“Our ability to reach unity in diversity will be the beauty and the test of our civilization.”—Mahatma Gandhi.*

There is no denying the overwhelming evidence on the importance of diversifying the pediatric nephrology workforce to better serve our patients. Additionally, diversity has been shown to enhance scientific advancement. Teams that include a broad array of backgrounds (gender, age, race, ethnicity, etc.) benefit from a broader network of ideas (50). Members of diverse groups also process information more deeply and thoughtfully than members of homogenous groups (51). Diverse scientific teams have also been demonstrated to publish more novel scientific research (51, 52). As such, this further supports why the field of pediatric nephrology would benefit from increased recruitment, training and retention of a diverse pipeline of physicians and physician-scientists. To achieve this, we must critically examine and address the barriers to low workforce diversity. O'Brien et al. present a 3-tiered approach to dismantling those barriers by targeting low applicant diversity, appointment bias, and departure bias (53).

Low applicant diversity stems from lack of role models which then leads to less engagement by URiM candidates into the pipeline. Recognizing the critical role of mentorship, various stakeholders in nephrology including the National Institutes of Diabetes and Digestive and Kidney Diseases (NIDDK), ASN, and ASPN have developed targeted nephrology trainee pipeline programs that provide undergraduate, medical and PhD students, and pediatric residents with early exposure to the field (54). While those programs are not explicitly targeted at URiM trainees, more recent efforts such as the ASN loan mitigation program are specifically designed to offer financial support to URiM candidates who are proportionally disadvantaged with rising costs incurred during entry into undergraduate and graduate medical education (53). Partnering with Historically Black Colleges and Universities (HBCU) to offer early acceptance programs into medical school is another attractive strategy that has been employed by some universities to diversify their pipeline (55).

Another unique aspect in pediatric nephrology is the large proportion of International Medical Graduates (IMGs) which constitute approximately 30%–55% of the US pediatric nephrology trainee workforce based on the trends tracked by the American Board of Pediatrics (ABP) since 2004 (56). The ability to retain IMGs from diverse cultural and racial-ethnic backgrounds post fellowship training can be a potent tool to improve diversity. Unfortunately, widespread lack of understanding of the unique immigration needs for IMGs have led to significant challenges in attaining satisfactory job positions post fellowship training, with almost 40% of pediatric nephrology IMGs reporting a negative perception of the national job market in the ASN 2019 Nephrology fellows survey (57). For division chiefs, working closely with their institutional legal affairs office and securing alternative non-NIH based research funding sources accessible to VISA holders and non-citizens (including professional society and foundation grants, listed in Table 2) can facilitate the retention of more IMGs in the field.

**Table 2:** Funding sources available to non-US citizen nephrology physician-scientists.

Name/organization	Key applicant criteria	Link
KidneyCure (formerly ASN Foundation)	• Must be working in North or Central America during the grant period • Must be an active member of ASN • Must hold an MD, PhD, or equivalent degree • Women and under-represented minorities in medicine encouraged to apply	https://www.kidneycure.org
American Society of Transplantation (AST)	• Work is to be performed in a North American research setting • Must have an MD, DO, PhD, DVM, PharmD or equivalent graduate degree, and have completed post-graduate training (residencies, post-doctoral fellowships, etc.) • Must be either: (a) a U.S., Canadian, or Mexican citizen; (b) a lawfully admitted permanent resident foreign national of the U.S., Canada, or Mexico with a valid visa during the awarded period; or (c) a foreign national admitted lawfully for residence in the U.S., Canada, or Mexico during the awarded period. J1 and H1B visa holders are eligible to apply	https://www.myast.org
Doris Duke Charitable Foundation	• Strongly encourages individuals from groups underrepresented in biomedical research to apply [including]… those who belong to populations whose exclusion from research based on their race, ethnicity, gender, disability, sexual orientation, or socioeconomic status has resulted in underrepresentation in the workforce; includes those who identify as: Blacks or African Americans, Hispanics or Latinos, American Indians or Alaska Natives, Native Hawaiians and other Pacific Islanders, women, individuals with disabilities, LGBTQ+, or having experienced limitations in access to science afforded by privilege (e.g., limited access to the knowledge, skill, and networks that facilitate enrollment in and graduation from a health professions school; or coming from a family with an annual income below a level based on low-income thresholds according to family size published by the U.S. Bureau of the Census)	https://www.ddcf.org
National Kidney Foundation (NKF)	• Must have completed training in nephrology prior to start of the grant award, and who intend to pursue research directly related to these areas, or • Must have trained in Internal Medicine or Pediatrics with subsequent Nephrology fellowship training • Have full-time appointment to a faculty position at a university, or an equivalent position as a scientist on the staff of a research-oriented institution, at the time funding begins • University or research-oriented institution must be in the United States	https://www.kidney.org/

*Appointment bias* refers to a perpetual culture of hiring practices that fail to recruit diverse candidates due to a combination of explicit and/or implicit bias, and lack of institutional commitment to equity, diversity and inclusion. The notion of “colorblind” hiring practices that aim to treat individuals as equally as possible, have unfortunately perpetuated pre-existing negative racial experiences of people of color, rejecting their heritage, and invalidating their unique perspectives. While most, if not all institutions have clear policies and escalation procedures to address explicit bias in the workplace, implicit bias is much more difficult to discern. *Implicit bias* refers to the unconscious attitude and beliefs that affect the feelings, behavior and judgment of a person towards another. On many occasions, the person exhibiting the implicit bias is unaware of the implications of their actions at that moment. However, those actions (or inactions) can negatively affect the quality of the interaction with the other party and potentially contribute to adverse recruitment outcomes. The medical field, and pediatrics specifically, is not immune to implicit bias in its many forms, be it gender bias, racial-ethnic bias, or others. Reference letters within medicine and medical education exhibit language discrepancies between men and women applicants with women applicants more likely to be described using communal adjectives, such as “delightful” or “compassionate”, while men applicants more likely to be described using agentic adjectives, such as “leader” or “exceptional” (58). Implicit racial bias among medical school admissions committee members have been implicated in recruitment of less diverse classes (59), while gender bias has been implicated in lower rates of promoting women to leadership roles (60).

In pediatrics specifically, “pro-white/anti-black” unconscious bias has been identified as a potential variable that has negatively affected the recruitment practices of academic faculty from minority backgrounds. Furthermore, even once hired, these biases have been shown to adversely impact the lived experience and retention of URiM pediatric academic faculty. Understanding one's own implicit biases is a first step to address this problem. This can be accomplished by taking an Implicit Association Test (IAT), though a collective institutional led effort to engage in meaningful discussions surrounding IAT test results is likely necessary for those individual efforts to reduce appointment bias. On an institutional level, adopting diverse recruitment strategies that include restructuring interview and search panels to include more diverse perspectives, using inclusive language in job advertisements, implicit bias staff training, and extending recruitment outreach beyond a typical “safe” pool of candidates used in the past, is key to mitigating appointment bias.

*Departure bias* describes the comparison of the diversity of the employees leaving an institution with the overall diversity of that institution (54). Moreover, it refers to the heightened risk of losing URiM candidates who have been successfully recruited into an organization due to lack of a supportive and nurturing work environment. In addition to the higher odds of facing explicit, and implicit bias in their day-to-day interactions, URiM candidates may be subject to frequent microaggressions in the workplace, leading to departure bias (61, 62). Microaggressions are commonplace daily verbal, behavioral or environmental slights, whether intentional or unintentional, that communicate hostile, derogatory, or negative attitudes toward stigmatized or culturally marginalized groups. Formal training on recognizing and addressing microaggressions in the workplace is lacking. Calardo et al. demonstrate the feasibility and effectiveness of deploying an anti-bias anti-racism curriculum for pediatric residents that is flexible and adaptable to virtual, in-person, and hybrid instruction to fill that need (63).

Other contributors to attrition over time include the assumed financial and quality of life burden exacted from years of required, costly, medical and specialty training (56, 64, 65). This is then compounded by the decision to continue in a trainee role (which commands a lower pay) for at least 3 years in order to complete a subspecialty training program. Upon completion of training, data on compensation and promotion provide further insight into the attrition rates. Results from the AAMC Faculty Salary Survey show female and racial-ethnic minority clinical science faculty with an MD degree are paid less than their male counterparts (48). Additionally, while women accounted for 51% of all medical school applicants in the 2018–2019 AAMC state of women in academic medicine report, only 25% achieved the rank of full professor, and less than 20% were department chairs or medical school deans (66). The inequity in pay, leadership, and promotion potential is a major demoralizing force that drives women and minority faculty out of academic medicine and discourages entry into the field. Leaders in academic medicine should act as cheerleaders, mentors, and sponsors to their junior women faculty and faculty of color to elevate their profile and facilitate their advancement along the academic medicine ladder. It is critical to advocate for pay equity and family friendly supportive work environments to reverse those trends.

Similar inclusive work policies are also crucial to supporting the Lesbian, Gay, Bisexual, and Transgender, and Queer (LGBTQ) members of the trainee pipeline and academic workforce; many of whom may hide or do not disclose their sexual or gender identity due to fear of discrimination (67). These additional responsibilities to retain and welcome diverse and minority trainees and faculty should not be placed solely on the shoulders of minority faculty “champions” in the name of diversity. Such practice, also known as the “minority tax”, leads to unrealistic expectations being placed on minority faculty, many of whom are in their early career stages, and ultimately leads to lower rates of promotion and consequently higher likelihood of departure from academic medicine (68).

It is evident that given the historical underpinnings of structural racism, dismantling inequities will require collective advocacy on a legislative level. Issues such as pediatric subspecialty loan repayment, NIH funding priorities, and payment parity with our adult nephrology counterparts are all critical to improving applicant diversity, and mitigating appointment and departure bias in pediatric nephrology. The ASPN has long supported those efforts by establishing the John E. Lewy Foundation (JELF) Advocacy Scholars program that serves as a mechanism to train and develop the next generation of effective pediatric nephrology advocates (69). The JELF scholars, along with other ASPN members, champion the society's public policy priorities towards a more equitable future for our patients and workforce members. The pursuit of leveling the playing field and achieving true “equity” in academic medicine will take time, however, it is reassuring to see the ABP, as the major certifying board for the specialty, and the AAP, the largest organization for pediatricians, both endorse wide ranging public policies to achieve that goal (70, 71).

**Figure 1:**
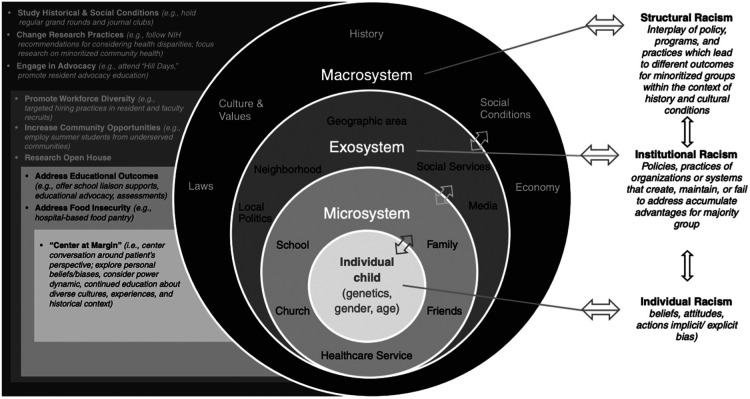
Illustration describing bronfenbrenner's socioecologic model of child development, which is affected by racism at every level, therefore requiring parallel interventions to combat racial disparities in health outcomes for children with kidney disease (72). Originally published by Dawson et al., 2022. Used with permission.

Finally, pediatric nephrology divisions require intentional strategic planning in regards to health equity. Dawson et al. describe the Nationwide Children's Hospital Kidney Health Advocacy and Community Engagement (KHACE) initiative, wherein multidisciplinary teams promote layered changes in the domains of research, education, engagement and policy within a socioecologic model of health (72). Action items include the study of historic conditions and SDOH; promoting health disparities research; engaging in advocacy; increasing workforce diversity; addressing educational outcomes; pursuing quality improvement initiatives to increase diversity amongst trainees; and centering care around the patient's perspective. The authors outline multiple tiers of interventions that address structural racism, institutional racism, and individual racism within a socioecological model (Figure 1).

## Conclusion

The pediatric nephrology care team must understand that race is a social construct with lasting and profound impacts on the minoritized, and that physicians should acknowledge social determinants of health along with understanding of pathophysiology, diagnosis, and treatment planning. Pediatric specialists must acknowledge the role racism contributes to disparate health outcomes *via* two main mechanisms: first, by enforcing a pervasive system of inequality through social determinants of health; and secondly, by generating a biologic impact on sympathetic nervous system activity, altered gene expression and altered hormonal metabolism (73). The impact of racism and bias on pediatric nephrology transplant and dialysis care should continue to be studied and addressed. We must prepare ourselves to identify and reduce systemic, institutional and individual bias and improve the diversity and sense of belonging within our field by tracking more accurately relevant demographic data on under-represented members of the medical field, and removing barriers to clinical and research support. Finally, we should adopt a strategic socioecologic and multidisciplinary approach to child health to address SDoH, along with primary care, research, social work and psychology colleagues. As stated by by Bignall and Crews, “racism is one of America's most enduring public health risks” (6), and it is the charge of the pediatric nephrology community to take an active role in dismantling it.
